# Accurate Identification of Cancerlectins through Hybrid Machine Learning Technology

**DOI:** 10.1155/2016/7604641

**Published:** 2016-07-13

**Authors:** Jieru Zhang, Ying Ju, Huijuan Lu, Ping Xuan, Quan Zou

**Affiliations:** ^1^School of Software, Tianjin University, Tianjin, China; ^2^School of Computer Science and Technology, Tianjin University, Tianjin, China; ^3^School of Information Science and Technology, Xiamen University, Xiamen, China; ^4^College of Information Engineering, China Jiliang University, Hangzhou, Zhejiang, China; ^5^School of Computer Science and Technology, Heilongjiang University, Harbin, China; ^6^State Key Laboratory of Medicinal Chemical Biology, NanKai University, Tianjin, China

## Abstract

Cancerlectins are cancer-related proteins that function as lectins. They have been identified through computational identification techniques, but these techniques have sometimes failed to identify proteins because of sequence diversity among the cancerlectins. Advanced machine learning identification methods, such as support vector machine and basic sequence features (*n*-gram), have also been used to identify cancerlectins. In this study, various protein fingerprint features and advanced classifiers, including ensemble learning techniques, were utilized to identify this group of proteins. We improved the prediction accuracy of the original feature extraction methods and classification algorithms by more than 10% on average. Our work provides a basis for the computational identification of cancerlectins and reveals the power of hybrid machine learning techniques in computational proteomics.

## 1. Introduction

Lectins, which can combine with sugars, are proteins that are produced and secreted by animal and plant cells. These proteins play a key role in cell-to-cell recognition and cellular adhesion, especially cellular interactive adhesion, because they contain many carbohydrate-combining sites. Cancerlectins are well-known lectins because of their source, sequences, binding site architecture, quaternary structure, and carbohydrate specificity. They participate in cancer-related processes, such as tumor cell differentiation, cancer cell monitoring, tumor tissue cell marking, and cancer metastasis.

Cancerlectins are typically identified through biological experiments, but these are costly and inefficient. As such, computational prediction approaches have been employed to verify novel cancerlectin protein sequences and to obtain cancerlectin candidates. Prediction accuracy is an important parameter, which when optimized can reduce the cost of computational prediction approaches. However, the accuracy rates of existing calculation and prediction methods are approximately 70%, which is unsatisfactory and thus should be improved. In the current study, we evaluated different feature extraction algorithms and classifiers to establish novel combinatorial machine learning strategy that can improve prediction accuracy.

Machine learning techniques instead of traditional sequence alignment methods, such as PSI-BLAST [1], HMMER [2], and HAlign [3], are often used to identify special proteins. Among these identification techniques, a support vector machine is the most common classifier used in computational proteomics, which involves various processes, such as classifying protein subfamilies [4–6], predicting protein structural classes [7], and identifying thermophilic proteins [8]. Random forest is also a common classifier that works via an ensemble learning strategy and performs well in protein fold recognition [9]. In addition to random forest, heterogeneous basic classifiers are combined to classify imbalances [10] and improve accuracy [11–13]. Bioinspired computing models and algorithms can also be used to design promising classifiers, such as spiking neural models [14–18] and evolutionary algorithms [19, 20]. All of these advanced machine learning methods have demonstrated satisfactory performance in cancerlectin identification, which has inspired us to combine different classifiers and feature extractors to optimize the accuracy of prediction. After comparing their efficiency and popularity, we chose the feature extraction methods and classification algorithms mentioned above to demonstrate the impact of machine learning on the field of cancerlectin identification.

Protein features are more important than machine learning techniques for achieving the high accuracy of protein prediction. The protein features most commonly used for feature extraction and classification are* k*-mer and Chou's PseACC representation [21, 22]. They perform well in a range of applications, including predicting protein submitochondrial locations [23], identifying Golgi-resident protein types [24], predicting microkit protein localization [25], and identifying bacteriophage virion proteins [26]. Position-specific scoring matrix is another good option, but obtaining it is time-consuming [16], which limits its application. In some instances, an analysis of protein secondary structures helps improve classification accuracy. However, the extraction of secondary structure features is time-consuming. Some studies have reduced the feature dimensions for biological sequences, such as by using the minimum Redundancy Maximum Relevance (mRMR) [27, 28] and Max-Relevance-Max-Distance (MRMD) [29]. Nevertheless, studies have yet to combine hybrid multisource features, which is the main contribution of the current work.

Related machine learning strategies have yet to be applied to distinguish cancerlectins from other lectins. Song and Pan [30] and Kumar et al. [31] employed SVM but obtained only approximately 70% accuracy. They tested basic sequence features and disregarded multiview feature combination. In addition Damodaran et al. [32] collected more than 500 cancerlectins, which are used here as a positive training set for machine learning. In this study, we aim to examine additional features and classifiers and to determine the optimal combination of hybrid machine learning techniques that can be used to achieve optimal accuracy in cancerlectin prediction.

## 2. Methods

### 2.1. Main Flow

Machine learning, which can be used in protein mapping, has evolved from computational learning theory and the field of pattern recognition. Algorithms are initially used to extract the features of amino acids; different classifiers are then employed to predict cancerlectins. Various machine learning algorithms, which are more efficient and accurate than traditional methods, such as SVM-Prot-based feature extraction algorithm [33] and libSimpleVote classifier, are also utilized to predict cancerlectins. Therefore, the efficient combination of feature extraction algorithms and classifiers has been extensively investigated.

Although numerous feature extraction algorithms and classifiers have been widely used and studied in the field of bioinformatics and in the computing industry, the combination of these two strategies has rarely been investigated and the development of efficient cancerlectin prediction methods has seldom been performed. Furthermore, the combination of feature extraction and classifiers has been disregarded by most researchers because of the large data requirement and laboriousness of the work.

In the current study, various feature extraction algorithms are investigated and different feature dimensions are combined to determine an accurate feature vector. Feature extraction results are then applied to different classifiers to predict cancerlectins. After performing these trials, the most accurate and efficient combination of feature extraction algorithm and classifier can be determined and the accuracy rate can be calculated. Thus, this study aims to evaluate existing feature extraction methods and to identify the appropriate dimensions that can be used to predict cancerlectins with the highest accuracy. An appropriate classifier is also necessary to predict cancerlectins. Other tools and methods are also utilized to reduce the dimension of feature vectors and to help improve the accuracy of prediction. The following concepts are considered in our study:

1. Various feature vector files in  .arff are calculated on the basis of a specific database (CancerLectinDB), and different dimensions are combined to create  .arff files.
2. Different classifiers are used to predict the mapping of cancerlectin, and different prediction results are compared in one table or graph to determine the most accurate prediction method.
3. Feature extraction and random forest based on Conjoint Triad and Pseudo-Amino Acid Composition are the most accurate combination of feature extraction algorithms and classifiers to predict cancerlectins.

**Figure 1:**
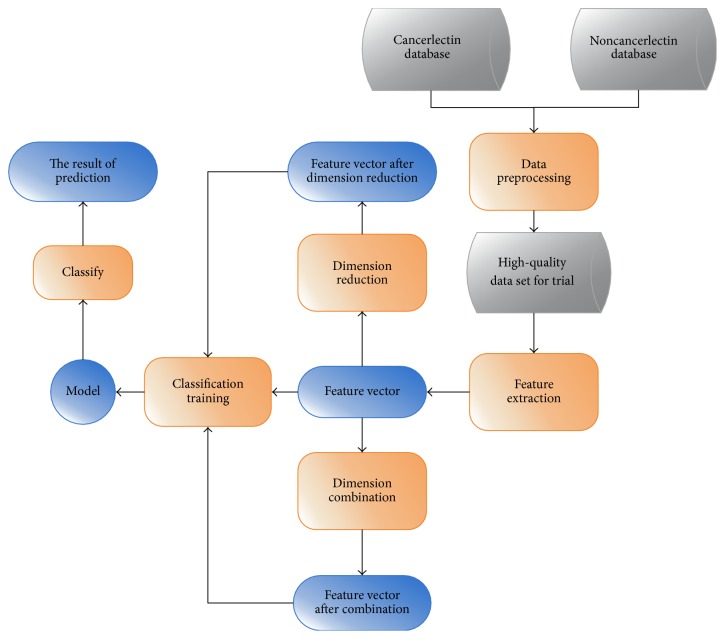
The main flow chart of the identification method of cancerlectin.

The main flow process is shown in Figure 1.

### 2.2. Data Preprocessing

CancerLectinDB, which is from a web server named CalecPred [30] and was provided by Professors Song and Pan, is used in this study to obtain high-quality data regarding cancerlectins. All of the training and the test sets were selected from this server as the data set in this work. Within the data set, 178 cancerlectins and 226 noncancerlectins are used as a training set and 20 other cancerlectins and noncancerlectins are utilized as a test set. In some feature extraction algorithms found in ProtrWeb [34], some cancerlectin and noncancerlectin sequences cannot be included because the protein sequence is too long to fit the methods; as such, we excluded these protein sequences to ensure an appropriate fit with the corresponding feature extraction methods.  Table 1 shows the number of lectins used in some feature extraction algorithms in ProtrWeb after the excluded data have been removed.

#### 2.2.1. Sequence Motifs Discovery

In order to clearly visualize the data, MEME [35] was used to analyze the conserved motifs among the cancerlectins. Because there is a limitation in the number of amino acids, we divided the set of cancerlectins into two groups. The five most significant conserved motifs of the first group are shown in Figure 5 and Table 5, and the motifs of the second group are shown in Figure 6 and Table 6.

#### 2.2.2. Training Set Balancing

There are 178 positive samples (cancerlectins) and 226 negative samples (noncancerlectins) in the training set. This inconsistency between the two groups could result in inaccurate results. In order to optimize the classification, we use the synthetic minority oversampling technique (SMOTE) [36] algorithm in Weka to supervise the instance. We also apply SMOTE to the training set of two main feature extraction methods: Conjoint Triad and Pseudo-Amino Acid Composition. The numbers of positive and negative samples before and after balancing are shown in Table 7. In addition, the comparisons before and after balancing the training set are shown in Table 8. We can see from Table 8 that, after balancing the positive and negative samples, the accuracy of cross-validation increases, but the accuracy of the method with the supplied test set decreases.

### 2.3. Feature Extraction Algorithm

#### 2.3.1. Conjoint Triad Feature

Conjoint Triad Feature (CTF) is a feature extraction algorithm used to obtain protein dimensions. It is based on neighbor relationships in protein sequences. This algorithm encodes each protein sequence by using a triad frequency distribution, which is extracted from a seven-letter reduced alphabet. It is also applied to formulate protein samples and perform predictions. CTF clusters 20 amino acids into seven classes [37] and regards any three consecutive amino acids among them as a single unit. A total of 343 dimensions of cancerlectin sequences are extracted by using the CTF algorithm. It transfers the file from  .csv format into  .arff format. These  .arff format files are then placed in some classifiers, such as random forests, for analysis and prediction.

A cancerlectin sequence is represented by *C* and is composed of *L* amino acids:

(1) $$\begin{array}{c} C=A_{1}A_{2}A_{3},\ldots ,A_{L}. \end{array}$$

We can include three amino acids in one group, as follows: *A*_1_ *A*_2_ *A*_3_, *A*_2_ *A*_3_ *A*_4_, *A*_3_ *A*_4_ *A*_5_, *A*_4_ *A*_5_ *A*_6_,…, *A*_*L*−3_ *A*_*L*−2_ *A*_*L*−1_, *A*_*L*−2_ *A*_*L*−1_ *A*_*L*_. The CTF of a cancerlectin is considered as the normalized frequency of these corresponding trimers in a sequence of a cancerlectin and is expressed as follows:

(2) $$\begin{array}{c} CTF={\left[\;F_{1},F_{2},F_{3},\ldots ,F_{k}\;\right]}^{T}, \end{array}$$

where *F*_*i*_ is the frequency of the three consecutive residues and *k* = 7^3^ = 343. Because the 20 kinds of amino acids can be divided into seven classes and we have three amino acids in one unit, for each unit, there can be 7 × 7 × 7 different combinations, so we finally obtain 343 dimensions [38].

#### 2.3.2. Pseudo-Amino Acid Composition

Pseudo-Amino Acid Composition (Pse-AAC) [39] is an approach incorporating contiguous local sequence-order information and global sequence-order information into the feature vector of a protein sequence. This approach can be used to obtain a feature vector with 50 dimensions. After some calculations are performed in ProtrWeb, the feature vector file in  .arff can be created. The feature extraction vectors can then be placed in classifiers to obtain prediction results.

*C* can be further expressed as follows:

(3) $$\begin{array}{c} C=A_{1}A_{2}A_{3},\ldots ,A_{L}. \end{array}$$

The Pse-AAC feature of a protein is defined as follows:

(4) $$\begin{array}{c} \text{Pse-AAC}={\left[\;F_{1},F_{2},F_{3},\ldots ,F_{k}\;\right]}^{T}, \end{array}$$

where *F*_*i*_ is the frequency of the amino acid calculated by the Pse-AAC algorithm and *k* = 50.

### 2.4. Classifier Selection and Tools

#### 2.4.1. Weka and Random Forest

Waikato Environment for Knowledge Analysis (Weka) is a well-known suite of machine learning software, which is used for data analysis and predictive modeling. In this study, Weka is used as a classifier. Among the options of Weka, “Classify” provides different modes of classifiers, such as random forest, ZeroR, KStar, and libSVM.

Random forests are used to obtain the average of multiple deep decision trees and are trained on different parts of the same training set to reduce variances. They are also considered a learning method for certain tasks such as classification and regression. Furthermore, random forests are used as a model for the rapid and efficient method of classification. This model applies bagging but uses a modified tree learning algorithm to select and split candidates during learning. In this method, different decision trees are determined for classification.

Weka also includes other test options, such as supplied test set, cross-validation, and percentage split. In this study, supplied test set and cross-validation are used to perform prediction. In the supplied test, training data and test set data should be provided for prediction. In the cross-validation, a single data set is split into a test data set and a training data set by using a specific algorithm.

#### 2.4.2. libSVM and Grid

libSVM [40] is an open-source machine learning library that implements the SMO algorithm for kernelized support vector machines and supports classification and regression; this library has been widely used to solve many tasks in bioinformatics [41, 42]. To apply this tool in our research, we download and install certain configuration files, especially Python. We execute all commands in a command line based on the runtime system of Python.

In this study, Grid was added to libSVM to tune parameters *c* and *g* and to enhance the accuracy of the prediction results. *c* and *g* are two training parameters provided by SVM with a Gaussian kernel function. Parameter *c* controls the overfitting of the model and parameter *g* controls the degree of nonlinearity of the model. *g* is inversely related to *c*, which represents the distribution around the statistical mean. Larger values of *c* will result in a model with low bias and high variance, and smaller *g* also corresponds to a model with low bias and high variance. Thus, the behavior of the kernel is less distributed or more nonlinear. These two parameters are determined by Grid search and cross-validation. The model with the highest estimated performance determines the selected training parameters. Then, these two parameters are used to predict libSVM to establish an SVM model and to obtain a more accurate prediction result. In the following section, the combinations of feature extraction and classifier for which the accuracy rate is >70% are reevaluated in libSVM.

## 3. Results and Discussion

### 3.1. Multidimension Combination Prediction

In this section, the feature extraction algorithms excluded from ProtrWeb are mainly investigated. These algorithms are referred to as multiple dimension combination prediction (MDCP) tools because their use involves different feature extraction methods and their combinations to obtain feature vectors and perform prediction. In the feature extraction part, different methods are employed to determine the vectors: 1-skip, 2-skip, 188-dimension feature extraction, 473-dimension feature extraction, and some algorithm combinations. In general, the 188-dimension feature extraction is based on physicochemical characteristics, and the* n*-skip algorithm is the same as a* k*-mer algorithm. In the classification part, the supplied test set and the cross-validation set are used for prediction.

**Figure 2:**
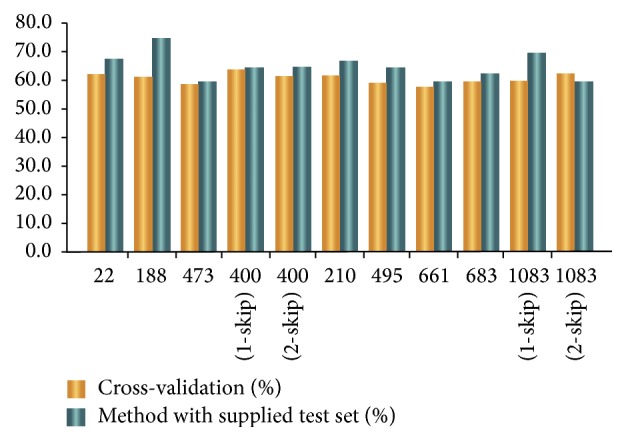
The accuracy rate of prediction in Part I.

After the combination of various dimensions and the conversion of file format, various  .arff files with different dimensions are obtained with a specific file head. We place the  .arff files into random forest classifiers in Weka for prediction.  Table 2 lists the exact dimensions of the algorithms.  Figure 2 shows the prediction results based on cross-validation and supplied test set validation. In Figure 2, 188-dimension feature extraction yields the highest accuracy rate of 75% when the supplied test set validation is applied.

### 3.2. ProtrWeb-Weka Prediction

In this section, the following algorithms provided by ProtrWeb are examined: Amino Acid Composition, Dipeptide Composition, Normalized Moreau-Broto Autocorrelation, Moran Autocorrelation, Conjoint Triad, Sequence-Order-Coupling Number, Quasi-Sequence-Order Descriptors, Pseudo-Amino Acid Composition, Amphiphilic Pseudo-Amino Acid Composition, Tripeptide Composition, and C/T/D. Conjoint Triad and Pseudo-Amino Acid Composition are among the most commonly used algorithms. Tripeptide Composition is characterized by 8000 dimensions, which are too numerous to calculate. C/T/D is an algorithm composed of three different methods and is too complicated for prediction. As such, these two algorithms are excluded, leaving the first 10 items in the list to be evaluated. The classifier provided by Weka is used for classification.

**Figure 3:**
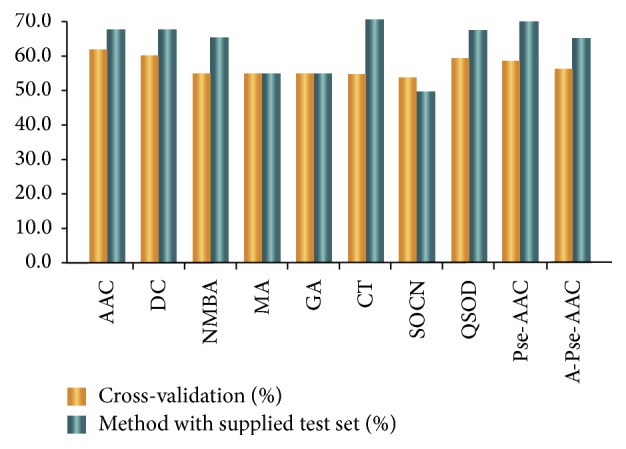
The accuracy rate of prediction in ProtrWeb.

**Figure 4:**
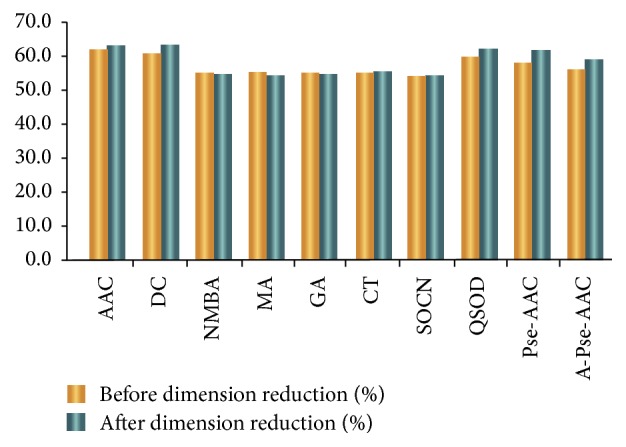
The accuracy rate of prediction before and after dimension reduction.

**Figure 5:**
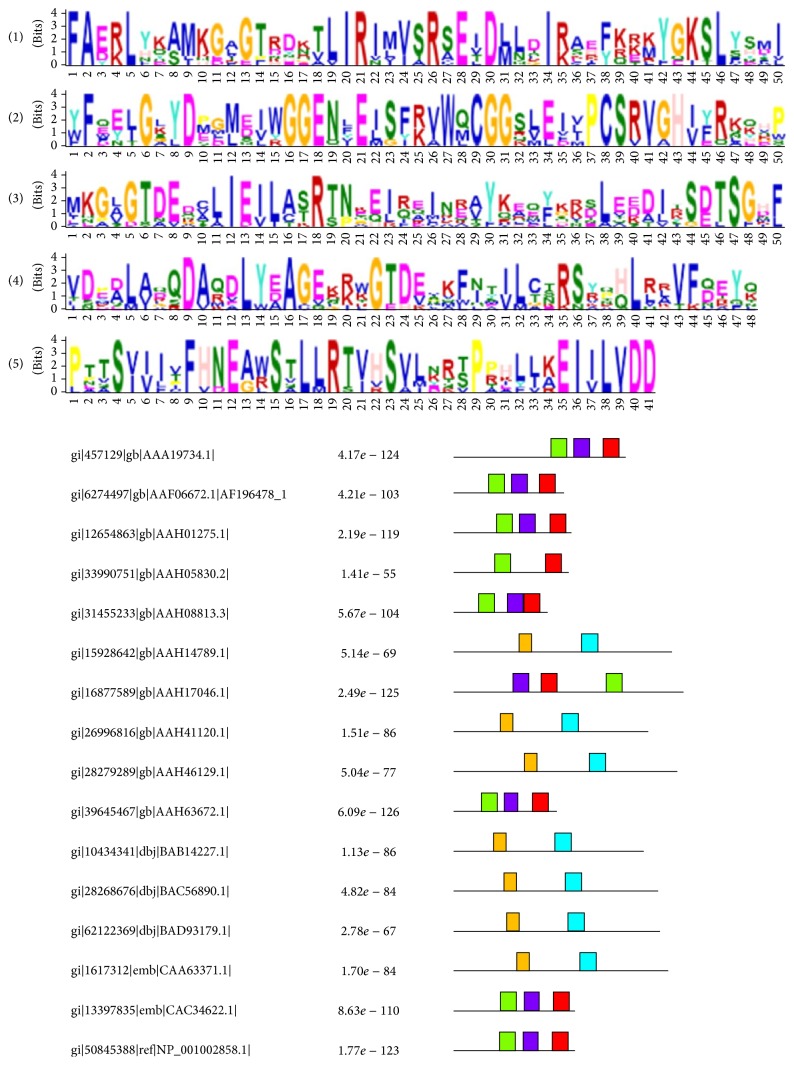
The most significant 5 conserved motifs of the first group.

**Figure 6:**
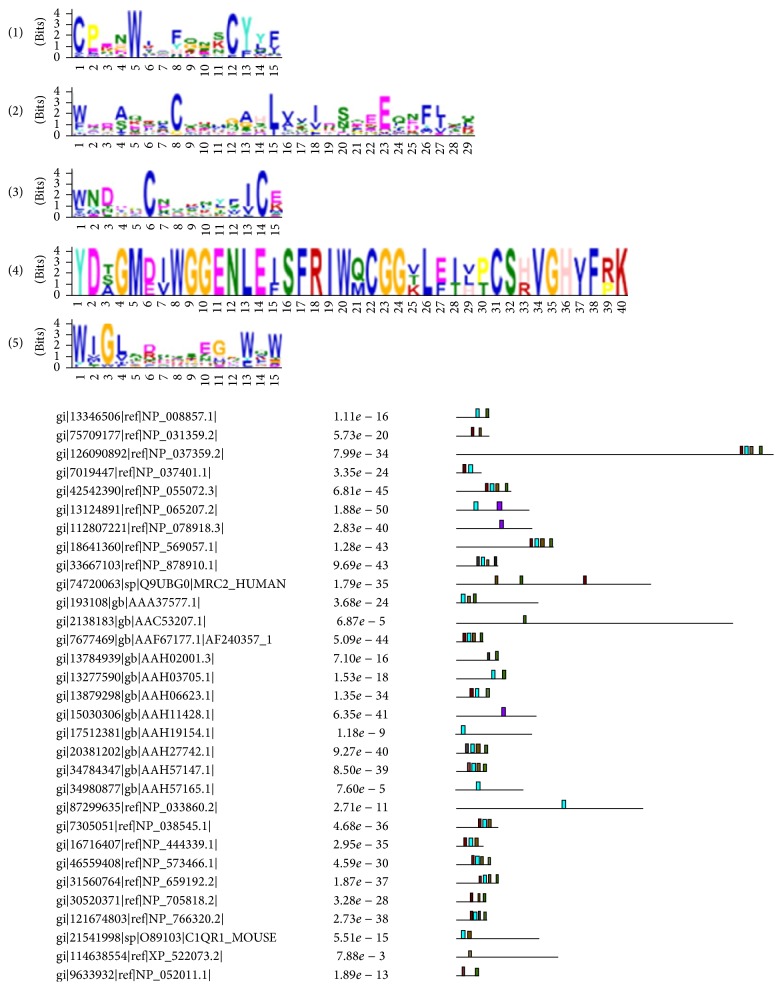
The most significant 5 conserved motifs of the second group.

Figure 3 illustrates the prediction results of cross-validation and supplied test set validation. We use the random forest as the classifier of extraction in Weka. The prediction accuracy rate of Conjoint Triad and Pseudo-Amino Acid Composition is 70%, which is higher than that of other algorithms. We also reduce the number of dimensions of the feature extraction algorithms by using MRMD [29].  Table 3 also lists the number of dimensions after they have been reduced.  Figure 4 reveals the accuracy rates of the prediction before and after the dimensions have been reduced.

### 3.3. ProtrWeb-libSVM Prediction

In this section, Conjoint Triad and Pseudo-Amino Acid Composition from ProtrWeb are included in libSVM for another cycle of evaluation. Considering the high accuracy rate of these two algorithms, which are classified by the classifiers in Weka, we aim to determine whether a more accurate prediction result can be obtained when a different classifier is used. Although Weka is a software suite into which various classification tools are integrated, some methods of prediction cannot be used with it. Hence, we employ libSVM for prediction. Each step in libSVM should be executed in the command line. For libSVM, the parameters *c* and *g* are set as the default values. Notably, *g* = 1/*k*, where *k* is the number of the cancerlectins.

Despite the advantages of libSVM, this method is still unable to achieve sufficient accuracy of prediction. Further studies should include additional parameters in the command line to obtain a prediction result that is close to the actual findings. To improve the predictive accuracy of this method, Grid is used to optimize the parameters *c* and *g*.  Table 4 summarizes the prediction results obtained in libSVM. The two methods fail to obtain high accuracy rates when classification is performed after these parameters have optimized.

## 4. Conclusions

Amino acid feature extraction and classification are major components of the prediction and classification of protein function. With advances in biology, medicine, and the biopharmaceutical industry, it should be possible to determine the positions of different proteins in cells. Although various amino acid feature extraction, fusion, and classification algorithms have been developed [43], they are independent of one another and are used for analyses in only one specific field of study. The combination of the two algorithms of feature extraction and classification has rarely been investigated and efficient methods for protein function prediction have seldom been developed. In this study, we comprehensively considered the two algorithms of feature extraction and classification in terms of their data set and basic logic. In this way, we determined the optimal strategy for combining feature extraction algorithms and classifiers. Thus, we performed numerous experiments and trials involving different algorithms. After conducting a substantial number of tests, we proposed a prediction method of predicting protein function comprising feature extraction and random forest classification based on Conjoint Triad and Pseudo-Amino Acid Composition. By using this combination, the accuracy rate reached 70%, which is higher than those of other prediction methods.

Our newly proposed method can thus be used to identify cancerlectins with reasonably high accuracy. Several network-based computational methods have already been applied to identify oncogenes [44] or oncomiRNA [45]. In addition, advanced social network algorithms have helped to predict the relationship between diseases and miRNA [46, 47]. However, network-based methods involve similar computation methods between miRNAs [48] or genes [49]. Information on interactions involving lncRNA [50, 51] and cell death [52] systems can improve prediction of the relationship between RNA and diseases. As another example of network constructing, a random walk [53] technique has been commonly employed to construct networks and predict unknown relationships. Nevertheless, network-based methods have been disregarded in cancerlectin identification. It is suggested that network features should be considered to improve classification accuracy. Further studies should also focus on the combination of network-based methods and classification techniques. Moreover, big data technologies, including Mahout and Hadoop, could be utilized to cope with large-scale data [54].

## Figures and Tables

**Table 1:** The number of pieces of data used in the ProtrWeb.

	Train set		Test set	
	Cancerlectin	Noncancerlectin	Cancerlectin	Noncancerlectin
Amino Acid Composition	178	226	20	20
Dipeptide Composition	178	226	20	20
Normalized Moreau-Broto Autocorrelation	178	225	20	20
Moran Autocorrelation	178	226	20	20
Geary autocorrelation	178	226	20	20
Conjoint Triad	178	226	20	20
Sequence-Order-Coupling Number	178	225	20	20
Quasi-Sequence-Order Descriptors	178	225	20	20
Pseudo-Amino Acid Composition	178	225	20	20
Amphiphilic Pseudo-Amino Acid Composition	178	225	20	20

**Table 2:** Dimensions of feature extraction algorithms in Part I.

Mode	Dimension
Pse-in-one	22
188 dimensions	188
473 dimensions	473
1-skip	400
2-skip	400
188 dimensions + Pse-in-one	210
473 dimensions + Pse-in-one	495
473 dimensions + 188 dimensions	661
473 dimensions + 188 dimensions + Pse-in-one	683
473 dimensions + 188 dimensions + Pse-in-one + 1-skip	1083
473 dimensions + 188 dimensions + Pse-in-one + 2-skip	1083

**Table 3:** Dimensions of feature extraction algorithms in ProtrWeb.

Mode	Dimension	Dimension reduction
Amino Acid Composition	20	19
Dipeptide Composition	400	49
Normalized Moreau-Broto Autocorrelation	240	47
Moran Autocorrelation	240	43
Geary autocorrelation	240	220
Conjoint Triad	343	81
Sequence-Order-Coupling Number	60	17
Quasi-Sequence-Order Descriptors	100	42
Pseudo-Amino Acid Composition	50	23
Amphiphilic Pseudo-Amino Acid Composition	80	15

**Table 4:** The prediction results of libSVM.

Mode	libSVM (%)	libSVM + Grid (%)
Conjoint Triad	55.9406	81.1881
Pseudo-Amino Acid Composition	86.1042	70.9677

**Table 5:** The 5 most significant conserved motifs of the first group.

Motif	Width	*E* value	Best possible match
1	50	1.3*e* − 157	FA[ED][RK]L[YH][KQ][AS]MKG[AL]GT[RD]D[KN][TV]LIRI[ML] [VI]SR[SA]E[ITV]D[LM][LN]DI[RK][AS][EH][FY][KQR][KRE][KM] YGKSL[YS][SH][MD]I

2	50	3.3*e* − 131	[YW]F[EQ][EY][LI]G[KL]YD[EMP]G[ML][ED][IV]WGGEN[FL]E [IL]SF[RK]VW[QM]CGGS[LV]EI[ILV]PCSRVGH[IV][FY]RK[KQ]HP

3	50	6.2*e* − 105	MKG[ALV]GTDED[CAV]LIE[IV]L[AC][ST]R[TS][NP][EK][EQ][IL] [RQ][EAQ]IN[ER][AV]Y[KQ][EA][QE][FY][KG][KR][DS]LE[DEK] [DA][IL][KRT]S[DE]TSG[HD][FL]

4	50	1.6*e* − 089	VD[EP][AD]L[AV][DQ]QDA[QR]DLY[EAD]AGEK[RK][WK]GTD [EV]XKF[IN]T[IV]L[CT][NST]RS[YR][PQ][HQ]L[RL][ALR]VF[DQ] EY[QK]

5	41	2.1*e* − 086	PTTS[VI][IV]I[TV]FHNE[AG][WR]STLLRT[VI]HSVL[KN]R[ST]P [PR]HL[LI][KA]EI[IV]LVDD

**Table 6:** The 5 most significant conserved motifs of the second group.

Motif	Width	*E* value	Best possible match
1	15	8.2*e* − 066	CPENWIX[FY][GQ]N[KS]CY[YL]F

2	29	2.1*e* − 071	[WF]XD[AS][QEK]XXCXXXG[AG]HL[VA][VS][IV]D[SN]XEEQ [NDE]F[LI]QQ

3	15	2.6*e* − 034	WNDXXC[ND]XK[LN][YL][FS][IV]C[EK]

4	40	7.7*e* − 033	YD[AST]GM[DE][IV]WGGENLE[IF]SFRIW[QM]CGG[KTV]L [EF][IT][HLV][PT]CS[HR]VGH[VI]F[RP]K

5	15	1.6*e* − 030	WIG[LV]S[DR]XXSEGXWQW

**Table 7:** The numbers of positive and negative samples of training set.

	Before balancing			After balancing		
	Cancerlectin	Noncancerlectin	Total	Cancerlectin	Noncancerlectin	Total
Conjoint Triad	178	226	404	356	226	582
Pseudo-Amino Acid Composition	178	225	403	356	225	581

**Table 8:** The comparisons before and after balancing the training set.

	Before balancing		After balancing	
	Cross-validation	Method with supplied test set	Cross-validation	Method with supplied test set
Conjoint Triad	54.9505%	70%	71.134%	67.5%
Pseudo-Amino Acid Composition	57.8164%	70%	72.4613%	67.5%
